# The uncertain fate of a wandering appendicolith: personal experience and literature review

**DOI:** 10.3389/fsurg.2025.1646625

**Published:** 2025-09-17

**Authors:** Andrea Cavallaro, Antonio Zanghì, Paolo Di Mattia, Filippo Sanfilippo, Luigi La Via, Giordana Riccioli, Alessandro Cappellani, Kenya Tiralongo

**Affiliations:** ^1^ General Surgery III, Department of General Surgery and Medical-Surgical Specialties, University of Catania, AOU Policlinico “G. Rodolico—San Marco”, Catania, Italy; ^2^ Department of General Surgery and Medical-Surgical Specialties, Chief ChiSMaCoTA Research Center, AOU Policlinico “G. Rodolico—San Marco”, Catania, Italy; ^3^ General Surgery, Umberto I Hospital, University KORE, Enna, Italy; ^4^ Department of Anaesthesia and Intensive Care, AOU Policlinico “G. Rodolico—San Marco”, Catania, Italy; ^5^ School of Anaesthesia and Intensive Care, University of Catania, AOU Policlinico “G. Rodolico—San Marco”, Catania, Italy

**Keywords:** retained appendicolith, hepatic abscess, laparoscopic appendectomy, recurrent infection, case report

## Abstract

**Introduction:**

Retained appendicoliths are an uncommon but clinically relevant complication of appendectomy, particularly in cases of perforated appendicitis. Migration of the appendicolith into the peritoneal cavity or liver may lead to persistent or recurrent abscess formation.

**Case description:**

We present the case of a 29-year-old male with recurrent hepatic abscesses following laparoscopic appendectomy. Initial CT and MRI imaging revealed calcified components within the liver consistent with a migrated appendicolith. Despite percutaneous drainage and antibiotic therapy, the patient experienced relapse. Definitive resolution was achieved through laparoscopic hepatic resection and removal of the retained appendicolith.

**Discussion:**

This case highlights the diagnostic challenges and potential complications associated with retained appendicoliths. Imaging plays a crucial role in identification and management. While conservative approaches may be attempted, surgical retrieval is often required to prevent recurrent infections.

**Conclusion:**

Retained appendicoliths should be considered in the differential diagnosis of hepatic abscesses following appendectomy. Timely diagnosis and complete removal are essential to avoid long-term morbidity.

## Introduction

Appendicoliths are small, calcified masses of hardened fecal material that form within the lumen of the appendix. Their development is primarily associated with fecal stagnation in the intestinal lumen, leading to the deposition of inorganic salts that gradually harden and calcify the material. Consequently, conditions such as constipation and reduced intestinal motility are considered key contributing factors to appendicolith formation.

Computed tomography (CT) can detect appendicoliths in up to 30% of cases. While appendicoliths do not directly cause appendicitis, their presence has been linked to an increased risk of treatment failure in nonoperative management of acute appendicitis (1–4).

One uncommon yet significant complication of appendectomy is the retention of an appendicolith. This can occur when an appendicolith is extruded from the appendix through a perforation or is inadvertently left behind during surgery. Retained appendicoliths pose a risk of migration into the peritoneal cavity, retroperitoneum, or even the thoracic cavity, where they may serve as a persistent source of infection or lead to abscess formation (5–9).

This report presents a case of a patient with a history of recurrent liver abscesses.

This case highlights the clinical significance of retained appendicoliths as a potential source of recurrent infections and underscores the importance of thorough intraoperative removal to prevent complications.

## Material and method

This case report describes a 29-year-old male patient who presented with abdominal pain and recurrent fever and was diagnosed with a multiloculated hepatic abscess following a recent laparoscopic appendectomy.

Initial imaging (CT scan), performed in the Emergency Department, revealed an abscess (60 × 53 × 90 mm) in the VI/VII hepatic segments with necrotic components, air bubbles, and several grossly hyperdense calcified elements (the largest measuring approximately 13 mm in diameter).

**Figure 1:**
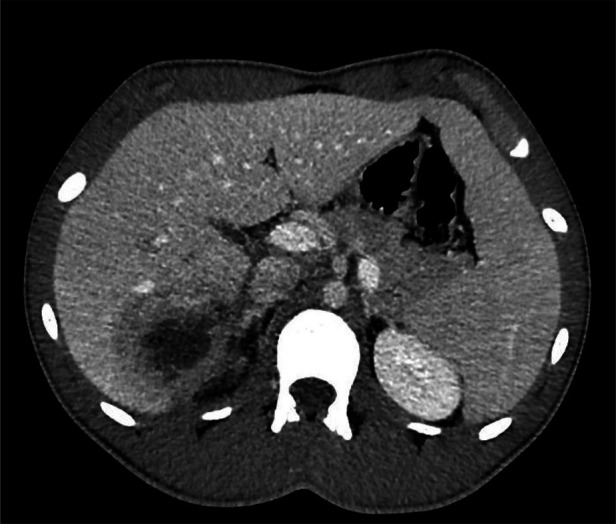
CT scan: Abscess (60 × 53 × 90 mm) in hepatic segments S6/S7, with necrotic components and intralesional air bubbles.

**Figure 2:**
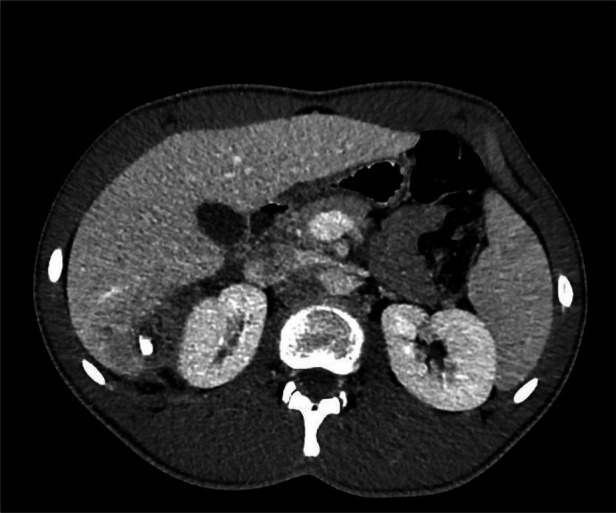
CT scan: Abscess in S6/S7 containing a hyperdense calcified element.

**Figure 3:**
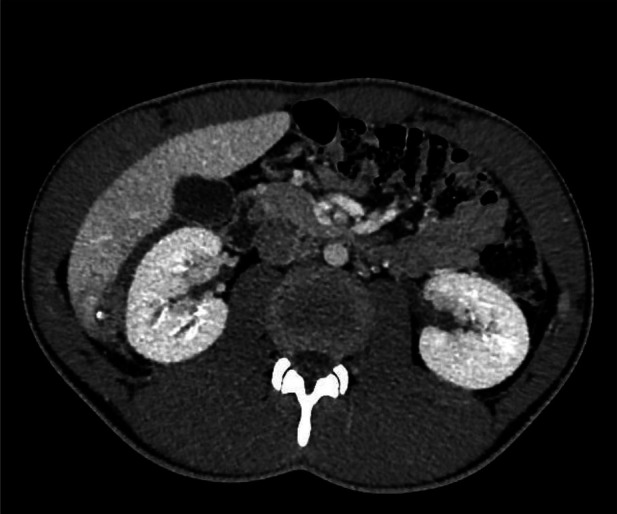
CT scan: Abscess in S6/S7 with an additional hyperdense calcified element.

This area demonstrated contrast-enhancing walls, suggesting a multiloculated hepatic abscess (Figures 1–3).

The patient underwent percutaneous drainage (8 Fr) under ultrasound guidance by the interventional radiology team segment with the microbiological exam of the purulent drainage identifying Escherichia coli.

The patient was discharged after one week in good health, afebrile for several days, asymptomatic, with a prescribed antibiotic regimen.

Despite initial clinical improvement and discharge, follow-up ultrasound imaging (two months after discharge) indicated residual abscess formation with multiple evolving collections.

**Figure 4:**
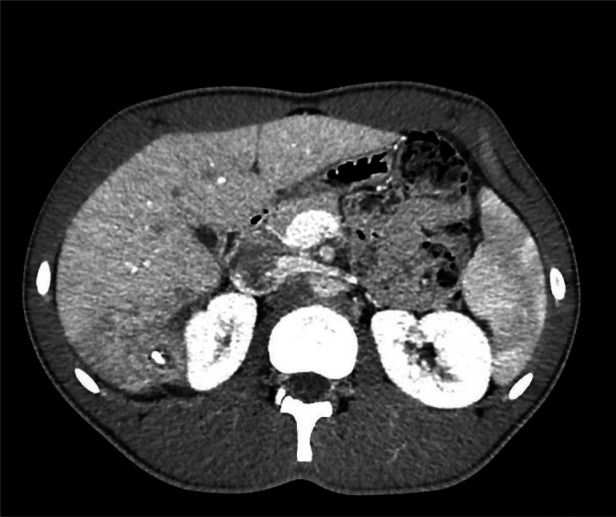
CT scan (after 4 months): Persistent but reduced round collection in S6, showing peripheral enhancement.

**Figure 5:**
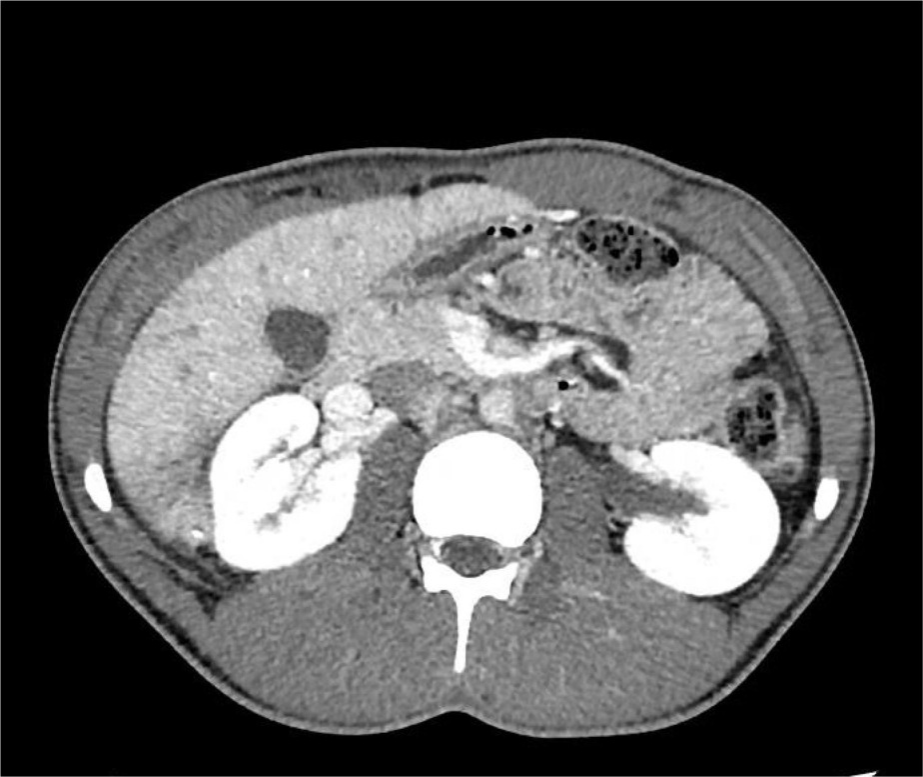
Abdominal MRI (after 4 months): Organized collection in S6 with wall thickening. The solid rim (high T2 signal intensity) demonstrates marked post-contrast enhancement.

Four months after the discharge, because of recurrent fever, the patient was admitted in the Emergency department. The patient underwent CT scan: in the VI hepatic segment, a round fluid/supra-fluid collection with peripheral enhancement measuring approximately 24 mm in maximum axial diameter was observed. A second collection with similar densitometric characteristics and a maximum diameter of approximately 7 mm is noted caudally in the same segment. Another area with similar features, approximately 12 mm in maximum axial diameter, is “identified at the hepatic dome”. (Figures 4, 5)

To better clarify the etiology of the process, an MRI was performed: an organized collection in SVI measuring approximately 52 × 24 × 32 mm, with a parietal thickness of about 7 mm and non-contrast-enhanced content showing high T2 signal and intermediate T1 signal with a concurrent fluid-fluid level. A lithiasic concretion with signal void in both weightings, appearing calcific on CT, is identified within. The solid rim, showing high T2 signal, exhibits marked post-contrast enhancement and a linear outer rim with delayed contrast uptake, resembling a pseudocapsule. This structure has significantly decreased in volume compared to the previous CT scan.

**Figure 6:**
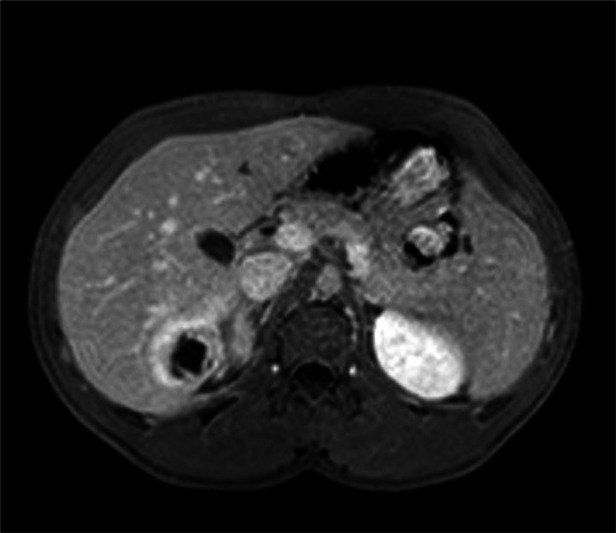
CT scan: Second collection located caudally within the same segment (S6).

Another similar lesion measuring approximately 18 mm is noted subdiaphragmatically in segment VIII (Figure 6). Blood cultures were negative for bacterial growth.

Following consultation with interventional radiologists, no indication for further drainage was found. Consequently, the patient, now afebrile, was discharged with prolonged antibiotic therapy (Piperacillin-tazobactam, 10 days) and scheduled for repeated outpatient follow-ups.

After two months, the recurrence of intermittent fever supported the indication for surgical intervention. The patient underwent laparoscopic hepatic resection of the abscess wall from segment VI and fenestration of the satellite lesion in segment VIII.

The post-operative course was uneventful, with no complications.

## Discussion

Liver abscesses can develop through several pathways:

• Hematogenous spread of infection via the portal vein or hepatic arteries.
• Biliary dissemination due to ascending cholangitis or cholecystitis with gallbladder infection.
• Direct inoculation following penetrating or iatrogenic trauma related to medical procedures.

Risk factors for liver abscesses include intra-abdominal infections, necrotizing enterocolitis, trauma, interventional procedures involving the biliary tract, liver cirrhosis, bacteremia, biliary diseases, chronic inflammatory bowel diseases, pancreatic disorders, gastroduodenal ulcerative diseases, and immunosuppression.

Radiology plays a crucial role not only in diagnosis but also in management, particularly through percutaneous drainage of liver abscesses, which can be performed under ultrasound or CT guidance. Surgical intervention is generally reserved for cases where percutaneous drainage is not feasible or proves ineffective.

A thorough review of the literature highlights that intra-abdominal abscesses following appendectomy, particularly after perforated appendicitis, are among the most common complications. Prospective studies have reported an abscess rate of up to 20% in patients presenting with a continuous defect in the appendix or an appendicolith detected intraoperatively (1).

Appendicoliths form within the appendix due to the accumulation of fecal matter and inorganic salts and typically appear as calcified masses within the appendiceal lumen. In rare cases, a coprolith may originate from the colon (especially the sigmoid colon), rectum, or, exceptionally, the small intestine (1, 2).

Laparoscopic appendectomy is the preferred surgical approach for treating acute appendicitis and draining periappendiceal abscesses (3). This technique offers the advantage of allowing thorough peritoneal cavity exploration and enables drainage of abscesses that are not accessible via percutaneous methods.

However, laparoscopic dissection can be particularly challenging in the presence of adhesions or a concurrent phlegmon. Moreover, the extensive irrigation required during laparoscopic drainage carries a risk of recurrent abscess formation and the potential migration of appendicoliths (fecaliths/coprolites), with uncertain outcomes (4).

Retained intra-abdominal coproliths may result from appendiceal rupture prior to surgery or failure to remove them during the procedure. Migrated appendicoliths can lodge in nearby organs either preoperatively or during surgery, subsequently leading to delayed infections (1–4).

The clinical manifestations of retained or migrated appendicoliths vary widely, including intra-abdominal abscesses, delayed wound healing, fistula formation, appendicular torsion, and stump appendicitis.

Unusual cases described in the literature include recurrent iliopsoas abscesses due to late-onset appendicolith migration (5), gluteal abscesses with intra-abdominal extension (6), subpulmonary abscesses, and pneumonia following appendectomy for perforated appendicitis (7).

Moreover, a pelvic abscess mimicking a malignant urachal tumor was reported in a 41-year-old male two years post-laparoscopic appendectomy (8), and a tubo-ovarian abscess was documented as a consequence of appendicolith migration into the Fallopian tube (9).

Only a few cases of liver abscesses resulting from appendicolith migration have been documented. Treatment options include percutaneous, open, or laparoscopic drainage of the abscess, often combined with retrieval of the coprolith, as antibiotics and drainage alone may be insufficient. Surgical removal of an intrahepatic fecalith is not always required; several reports describe successful percutaneous extraction using interventional radiology techniques, such as Dormia baskets or adaptations of endourological and nephrolithotomy methods (10–14).

Parasurgical procedures and extraperitoneal approaches have been proposed to minimize the intra-abdominal complications associated with laparoscopic or open surgery, such as infection spread, bowel injury, and adhesions. These techniques allow for abscess drainage while preserving peritoneal integrity (10–14).

A recent review of the literature identified 11 cases of liver abscesses secondary to migrated appendicoliths across 10 reports (15).

All patients had previously undergone appendectomy for perforated appendicitis, and the appendicolith migration was presumed to have occurred due to the perforation. The patients, aged between 6 and 37 years, developed signs of inflammation or sepsis between 7 days and 2 years post-appendectomy. All were diagnosed with perihepatic abscesses caused by migrated appendicoliths. Among the cases reviewed:

• One patient was successfully managed with antibiotics alone, without abscess drainage.
• Two patients underwent percutaneous abscess drainage, followed by percutaneous appendicolith extraction weeks later.
• One patient had successful percutaneous drainage without appendicolith retrieval.
• Two patients required percutaneous drainage followed by surgical appendicolith removal.
• Three patients underwent laparoscopic drainage with appendicolith retrieval, while one required a second laparoscopic procedure for an additional appendicolith.
• Two patients underwent laparotomy with abscess drainage and appendicolith removal.

The authors emphasize the importance of appendicolith retrieval, as abscess drainage alone is a known risk factor for persistent or recurrent infections (15). Every effort should be made during appendectomy to identify and remove any dislodged appendicoliths to prevent future abscess formation and other complications (3, 15, 16).

Conversely, some reports highlight the efficacy of percutaneous drainage combined with intravenous antibiotics, with no recurrence of abscesses over follow-up periods exceeding one year. Certain authors advocate for conservative management of appendicoliths, considering it a safer alternative to surgical retrieval due to the potential complications associated with surgery (17–19). Sheikh S. et al. reported successful outcomes in 4 out of 6 patients undergoing percutaneous drainage, suggesting that, given its minimally invasive nature and potential effectiveness, catheter drainage should be considered as an initial approach before resorting to surgical intervention (1).

## Conclusion

Our case highlights the importance of vigilant post-operative surveillance in patients with persistent abdominal symptoms and the potential for hepatic abscesses as a complication of intra-abdominal infections. Although rare, this post-appendicitis complication should be strongly suspected in patients with intra-abdominal or hepatic abscesses who have a history of appendicitis, particularly if it was complicated. Delayed identification of a migrated appendicolith may lead to recurrent abscesses, sepsis, empyema, or peritonitis. Management options for abscesses secondary to appendicolith migration include percutaneous, laparoscopic, or conventional open surgery, but complete retrieval of the appendicolith is crucial to prevent recurrence. Awareness of this phenomenon is essential for both radiologists and surgeons to minimize complications and reduce the risk of repeated hospital admissions.
